# Prevalence, risk, and outcomes of venous thromboembolic events in kidney transplant recipients: a nested case-control study

**DOI:** 10.1080/0886022X.2022.2161395

**Published:** 2023-01-23

**Authors:** Vinant Bhargava, Priti Meena, Anil Kumar Bhalla, Devinder Singh Rana, Ashwani Gupta, Manish Malik, Anurag Gupta, Vaibhav Tiwari

**Affiliations:** a Institute of Renal Science, Sir Gangaram Hospital, New Delhi, India; b All India Institute of Medical Sciences, Bhubaneswar, India

**Keywords:** Chemoprophylaxis, deep vein thrombosis, kidney transplant recipient, venous thromboembolic events

## Abstract

**Introduction:**

Thromboembolism is more common in kidney transplant recipients (KTRs) than in the general population. Studies evaluating arterial and venous thromboembolism (VTE) in KTRs are scarce and the magnitude and risk factors are mostly undefined.

**Methods:**

A nested control study was conducted from January 1, 2007, to December 31, 2019. Adult KTRs who were detected to have VTE events during this period were included. The primary outcome was to assess the prevalence of VTE in this population. Secondary outcomes were the assessment of the time to occurrence of the thromboembolic events after transplantation and assessing the risk factors and patient survival. For each subject studied, 4 controls were matched from the data set.

**Results:**

Amongst 2158 patients, 97 (4.5%) were found to have VTE. The median follow-up time was 3.9 years (6–156 months). A total of 101 VTE events were recorded. The most common site of VTE was the lower limb deep vein thrombosis in 79 patients (0.03%)).In multivariate Cox regression analysis, serum creatinine of more than 3 mg/dl [HR 1.30, 95% CI (1.03–1.38)] was independently associated with increased VTE risk. Patients who developed a VTE had higher mortality as compared to patients who did not develop VTE. No increased risk of graft failure was found in VTE patients.

**Conclusion:**

This study suggests that kidney transplantation surgery is a moderate risk factor for VTE, and VTE is associated with higher morbidity and mortality. However, prospective studies are needed to establish a definite role of VTE in outcomes in KTRs.

## Introduction

Kidney transplantation (KT) undoubtedly offers superior survival and a better quality of life for patients suffering from end-stage kidney disease (ESKD); however, these benefits are sometimes complicated by infections, side effects of immunosuppressive agents, rejections, cardiovascular complications, and rarely thrombotic complications [1]. Kidney transplant recipients (KTRs) are predisposed to hypercoagulation, resulting in a higher incidence of venous thromboembolism (VTE) than the general population [2]. The hypercoagulable state in KTRs is multifactorial; some of the risk factors are common in the general population, while others are specifically related to KT. In KTRs, previously recognized risk factors for VTE are old age, hypercoagulable state, malignancy, and sirolimus therapy. VTE during the early post-transplant period is mostly related to surgical procedures [3]. The literature suggests a higher recurrence rate of VTE after the first episode following the cessation of oral anticoagulant therapy, which further increases morbidity and mortality in KTRs [4]. The prevalence and implications of VTE on morbidity and mortality in KTRs are not well described in the literature. Moreover, there is a paucity of data concerning the management of VTE, including drug of choice, optimal duration, and secondary thromboprophylaxis. Herein, we aim to study the frequency of thromboembolic events and analyze the risk factors and outcomes of VTE in KTRs.

## Methods

The study was conducted in a tertiary care provider hospital with a yearly 300 kidney transplants. The study period was from January 1, 2007, to December 31, 2019. The inclusion criteria of participants were KTRs of age 18 years and older, who were detected to have VTE events in the study period. These patients were followed up from the date of a VTE episode until April 30, 2021 or until death. These patients were followed-up even if they developed graft failure, were initiated on dialysis, or underwent another transplant. Patients aged <18 years, who had received multi-organ transplants were excluded from the study. The data was collected from the electronic medical records of the hospital. The study was reviewed and approved by the institutional review board of the hospital. (EC/06/21/1943)

The observed data included the patient’s demographics, etiology of ESKD, immunosuppressive medication, graft function, comorbidities, thromboembolism prophylaxis, and the timing and site of VTE. Proteinuria and serum levels of creatinine were measured at the time of enrollment. Testing for thrombophilia profile was not included in the pre-transplant work of the patients since it was not a standard of care (as per institutional policy) unless there was a clinical suspicion of pro-thrombotic disorder such as recurrent thrombosis of vascular access or a prior history of VTE episode. The database was searched for the presence of antithrombin, protein C and S deficiency, G20210A prothrombin gene variant, factor V Leiden mutation, serum homocysteine levels, and antiphospholipid antibody (APLA) profile including anticardiolipin antibodies, anti-beta-2-glycoprotein-I antibodies and lupus anticoagulants in cases who were detected to have VTE. None of the patients received anticoagulation for primary prophylaxis of VTE/stroke. It is not a policy of our institute to use the prophylactic LMWH, as patients are mobilized from day 3 following kidney transplant surgery.

## Definitions

The diagnosis of VTE was made on clinical suspicion which was then confirmed radiologically. Deep vein thrombosis (DVT) was confirmed with findings of abnormal compressibility along with persistent filling defect or the presence of thrombus in the lumen by a duplex ultrasonography. Pulmonary embolism (PE) was confirmed using either ventilation/perfusion scanning or CT angiography.

Delayed graft function (DGF) was defined as the failure of a kidney allograft to function immediately after the transplant surgery or the need for dialysis within one week. Acute rejection was defined when biopsy-proven cases were given anti-rejection therapy. We defined graft failure as loss of graft function requiring dialysis initiation (death censored).

The present study is a nested case-control study. We defined cases as the patients having the first VTE event (index date). The selection of controls was done randomly as the KTRs with no VTE. For each case, 4 controls were matched for sex, age (±3 years), co-morbidities, body mass index (BMI), etiology of ESKD, dialysis modality before transplantation, immunosuppressive agents, had the same month of transplantation and duration of follow-up after transplantation (± 3 months).

The study was approved by the Institutional Board Review and Ethical Committee of the hospital. The clinical and research activities being reported are consistent with the Principles of the Declaration of Istanbul as outlined in the ‘Declaration of Istanbul on Organ Trafficking and Transplant Tourism’.

## Immunosuppression protocol

All KTRs received immunosuppression therapy of tacrolimus (0.15 mg/kg/day in two divided doses), and mycophenolate mofetil (MMF) 1 g twice a day, started 2 days before kidney transplant (KT) surgery

On day 0 preoperatively induction therapy was given. Either monoclonal (basiliximab) or polyclonal antibodies ATG (Sanofi Genzyme ATG Thymoglobulin) were given according to the immunological risk profile of the patients. Methylprednisolone was used in all patients. Following KT, patients were maintained on a triple-drug regimen. The most common regimen used was tacrolimus + MMF + glucocorticoids. Other combinations of immunosuppression were cyclosporine/prednisolone/MMF. A few other KTRs received a combination of prednisolone, tacrolimus 0.15 mg/kg/day in divided doses, along with everolimus.

The high-risk KTRs with CMV serology (Donor+/Recipient-) and Recipient + who received induction therapy with ATG received prophylaxis with valganciclovir for six months. KTRs with CMV serology Recipient + were given valganciclovir for three months. All patients received trimethoprim/sulfamethoxazole (80/400 mg) daily for 6 months.

## Outcomes

The primary outcome was the estimation of the prevalence of VTE events in KTRs and the evaluation of the effect of VTE on patient survival. Secondary outcomes were the assessment of the timing of occurrence of VTE events following transplantation, determination of the risk factors, and estimation of the rate of recurrence after thromboprophylaxis withdrawal. The crude death-censored graft failure rate for all recipients was also estimated.

### Statistical analysis

Results were expressed as median and range due to their skewed distribution. All continuous variables were expressed as the mean of standard deviations, whereas categorical variables were presented as numbers and percentages. Continuous variables were analyzed using the independent t-test and Mann–Whitney test. Different risk factors of VTE were assessed using the Cox regression model. Fine-Gray subdistribution hazard models with ‘VTE as endpoint’ and ‘death without VTE’ as competing risk was considered and the hazard rates associated with the risk factors were determined. The two states were treated as terminal states. A new time variable (etime) was created indicating the time to the first progression to VTE, death without VTE or the last follow-up, along with the event variable describing the outcome. By using the Fine-Gray approach, the fitting was done in a two stages process: a) the first stage created a special data set using the Fine-Gray method, and b) second stage fitted the weighted Cox model to the data. The Cox model fit to the constructed data set yielded the Fine-Gray model for VTE as the endpoint and death without VTE as a competing risk. Cox proportional hazards regression models were used to estimate the hazard ratio (HR) with 95% CI to analyze patient survival, it was adjusted for co-morbidities. Cumulative patient survival was analyzed by Kaplan-Meier survival curve. Multiple Cox regression models were applied to estimate adjusted HR for the effect of VTE on graft survival. We have excluded the patients with graft thrombosis from the analysis as all of them were due to severe acute rejections. *p* < 0.05 was considered statistically significant. All statistical analyses were performed using IBM Spss Statistical Software Version 21 0, supplied by SPSS Inc.

## Results

### Study population

We included 2158 KTRs in this study. There were 101 VTE events leading to 0.0236 VTE per person-year or 23.6 events per 1000 person-years. 101 VTE events were detected in 97 (4.5%) patients; 4 patients had recurrent VTE events. All but two patients received a living donor kidney. The median follow-up time was 3.9 years (range 6–156 months). Of the 97 patients, 66 (68%) were male, the mean age was 50 ± 2.3 years and the median proteinuria (mg/day) was 136 (48–3800) in VTE group and 142 (50–4200) in non-VTE group. (baseline characteristics are presented in Table 1). As induction therapy, 74 patients received anti-thymocyte globulin and 8 patients received basiliximab, whereas 15 patients received no induction therapy. 73 patients were on steroids, tacrolimus, and mycophenolate mofetil (MMF) as maintenance immunosuppression, and 10 patients were on steroids, everolimus and MMF for maintenance immunosuppression. Three patients had a history of malignancy, and 10 patients were positive for cytomegalovirus (CMV). No patients were smokers, and four patients had a body mass index (BMI) >28 kg/m2.

**Table 1.** Characteristics of the recipients and the donors.

Characteristics	Cases (*n* = 97)	Controls (*n* = 388)	p-value
Age, years ± (SD)	50 years ± 2.3	48 years ± 2.8	0.24
Male gender, n (%)	66 (68)	252 (65)	0.43
Body mass index , Kg/m^2^± (SD)	22 ± 1.3	21 ± 2.5	NS
Median proteinuria, mg/day	136 (48–3800)	142 (50–4200)	0.13
Etiologies of ESKD, n (%)			
Diabetes mellitus	54 (55.6)	204 (52.4)	NS
Chronic Glomerulonephritis	14 (14.4)	70 (18.1)	NS
Chronic interstitial nephritis	9 (9.3)	38 (9.8)	NS
Hypertensive nephrosclerosis	12 (12.5)	53 (13.6)	NS
Undetermined	8 (8.3)	23 (6)	NS
Coronary artery disease, n (%)	35 (35.05)	151 (38.9)	0.38
Peripheral artery disease, n (%)	6 (6.2)	32 (8.3)	0.45
ABO-incompatible, n (%)	12 (12.3)	55 (14.2)	NS
Median time in dialysis, months	12 (4–36)	9 (5–32)	NS
Hemodialysis , n (%)	94 (96.9)	379 (97.6)	NS
Peritoneal dialysis, n (%)	3 (3.1)	7 (2.2)	NS
Cytomegalovirus infection, n (%)	10 (10.7)	46 (12)	0.35
Prior history of DVT, n (%)	3 (3.1)	8 (2.1)	0.25
History of malignancy, n (%)	3 (3.1)	15 (4)	0.26
Transplant factors			
Acute rejection episodes, n (%)	10 (10.3)	42 (11.8)	0.14
Delayed graft function, n (%)	4(4.2)	14 (3.6)	NS
HLA mismatch number (means ± SD)	3.8 ± 1.2	3.6 ± 1.8	NS
Donor characteristics			
Living donors n (%)	95 (97.9)	382 (98.4)	0.36
Donor Sex, male/female (%)	21.4/78.6	23.4/76.6	NS
Age donor (yr) mean (sd)	45.4 (10.2)	44.6 (13.1)	0.23
Immunosuppression			
Induction agent , n (%)	82 (84.5)	326 (84)	NS
Basiliximab, n (%)	8 (8.2)	24 (6.6)	NS
Anti- thymocyte globulin, n (%)	74 (76.2)	302 (77.8)	NS
Steroid + Mycophenolate Mofetil + Tacrolimus, n (%)	73 (75)	310 (80)	NS
Steroid + Mycophenolate Mofetil + Cyclosporine n (%)	14 (14.2)	54 (14)	NS
Steroid + Mycophenolate Mofetil + Everolimus, n (%)	10 (10.7)	31 (10.01)	NS

ESKD: end stage kidney disease; DVT: deep vein thrombosis.

**Table 2.** Site of VTE.

SITE	VTE EVENTS (*n* = 101)
Lower limb DVT	79
Upper limb DVT	5
Pulmonary embolism	6
Cerebral venous sinus thrombosis	2
Iliofemoral thrombosis	5
Graft vessel thrombosis	4

VTE: venous thromboembolism; DVT: deep vein thrombosis.

**Table 3.** Multivariate analysis of potential risk factors for venous thromboembolic events in kidney transplant recipient.

Analysis of potential risk factors for venous thromboembolic events in kidney transplant recipient				
	Univariate analysis		Multivariate analysis	
Variates	HR, (95%CI)	p-Value	HR, (95%CI)	p-Value
Age more than 65 years	12.7 (10.5–30.9)	0.29	1.08 (0.67–1.65)	0.78
Male sex	0.53 (0.39–1.36)	0.64	0.78 (0.79–1.25)	0.58
History of Malignancy	34.0 (18.8–50.2)	0.04	1.12 (0.98–1.54)	0.12
Diabetes mellitus	1.10 (1.06–1.37)	0.17	0.49 (0.31–0.86)	0.06
Body mass index (>25.0 Kg/m^2^)	26.9 (20.8–47.0)	0.72	1.08 (0.81–1.45)	0.614
Use of mTor inhibitors	17.0 (13.4–30.9)	0.66	1.14 (0.83–1.54)	0.42
Cytomegalovirus infection	2.1 (1.33–3.54)	0.03	1.22 (0.84–1.75)	0.25
Aetiologies of end-stage kidney disease	1.05 (1.03, 1.06)	0.76	1.06 (0.68–1.65)	0.81
Prior history of deep vein thrombosis	2.34 (1.5–5.28)	0.04	1.40 (0.9–1.72)	0.67
Peritoneal dialysis before transplantation	17.4 (13.4–31.2)	0.36	0.38 (0.12–1.22)	0.10
Creatinine more than 3 mg/dl	1.20 (1.11–1.23)	0.001	1.30 (1.03–1.38)	0.03

mTOR: mammalian target of rapamycin; HR: hazard ratio.

### Site and timing of venous thromboembolism

**Figure 1.**
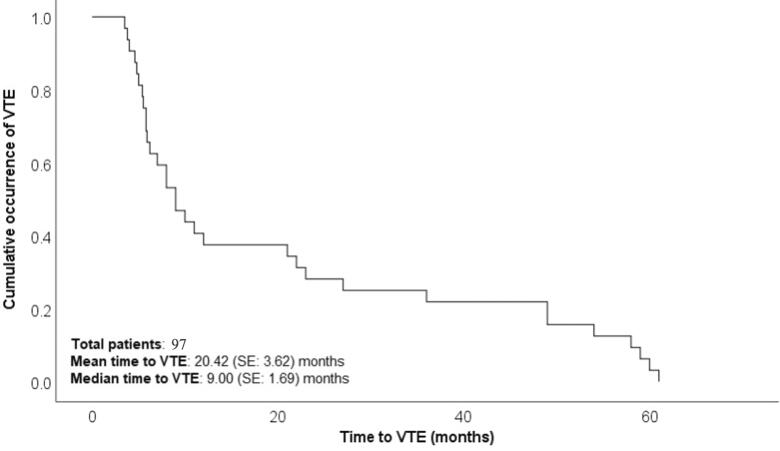
Kaplan-Meier plot showing cumulative VTE occurrences with time.

Out of 101 cases with VTE events, 79 were diagnosed with lower limb deep vein thrombosis (DVT), 5 cases with upper limb DVT, 6 cases with pulmonary embolism, 2 cases with cerebral venous sinus thrombosis, 5 cases with iliofemoral thrombosis, and 4 cases with graft vessel thrombosis (Table 2). The highest incidence of VTE was reported in the first 6 months following transplantation. The occurrence of VTE events was 36, 16, 22, and 27 at 0–6, 6–12, 12–48, and >48 months after transplantation, respectively. All four recurrent VTE events were lower limb DVT. Figure 1 presents a Kaplan–Meier plot showing cumulative VTE occurrence over time.

### Anticoagulation

All patients were initially treated with low-molecular-weight heparin (LMWH). In 68 patients, LMWH treatment was followed by warfarin; 12 patients received apixaban, 13 patients received rivaroxaban, and 8 patients received dabigatran. Bleeding-related complications during anticoagulation therapy were reported in four patients: two patients had gastrointestinal bleeding and another two patients had hematuria. All patients received oral anticoagulant treatment for a minimum of 6 months.

### Risk factors for VTE

All patients diagnosed with VTE were assessed for thrombophilia disorders. Nine patients had increased serum homocysteine levels. Factor V Leiden, antithrombin, protein C and S, prothrombin gene variants, anticardiolipin antibodies, anti-beta-2-glycoprotein-I antibodies, and lupus anticoagulant were found to be normal in all patients. Four cases of graft vessel thrombosis were diagnosed 3 months after transplantation, and all were associated with severe acute antibody-mediated rejection. All five cases of upper limb DVT had a history of tunneled central venous catheters for hemodialysis. Both cases of cerebral venous sinus thrombosis were the result of rhino-cerebral mucormycosis. Among the five cases of iliofemoral thrombosis, one was due to malignancy, one case had a history of immobilization due to femoral fracture followed by cast application, one case had CMV viremia, and no cause was found in the remaining two cases. Eight patients in the VTE group and 15 patients in the non-VTE group had nephrotic-range proteinuria (>3.5 gm/day), and no association with higher VTE risk was found. To identify the risk factors for VTE, univariate and multivariate Cox regression analyses were performed. Univariate analysis showed that prior history of DVT (HR 2.34, 95% CI 1.5–5.28, *p* = 0.04), CMV infection (HR 2.1, 95% CI 1.33–3.54, *p* = 0.03), history of malignancy (HR 34.0 95% CI (18.8–50.2 *p* = 0.04 and allograft dysfunction (serum creatinine >3 mg/dl) (HR 1.20, 95% CI 1.11–1.23, *p* = 0.001) were associated with increased VTE risk. However, following multivariate Cox regression analysis, serum creatinine >3 mg/dl (HR 1.30, 95% CI(1.03–1.38) *p* = 0.03 was independently associated with increased VTE risk when adjusted for age, sex, comorbidities, BMI, CMV infection, etiology of ESKD, and use of immunosuppressive medications. While using Fine-Gray subdistribution hazard model, it was evident that the hazard coefficients showed a very marginal change as compared to the conventional Cox proportional model. Only the age factor showed a significant effect on the hazard of VTE with a rate of 2.274 [95% CI: 1.069, 4.837] with a p-value of 0.033. The 95% CI of HR for sex and comorbidity were marginally narrower using the Fine-Gray approach as compared to the simple Cox model. Overall, the results were nearly consistent in both the models (Cox- regression and Fine-Gray subdistribution hazard model) (Table 3).

### Effect of VTE on patient survival

**Figure 2.**
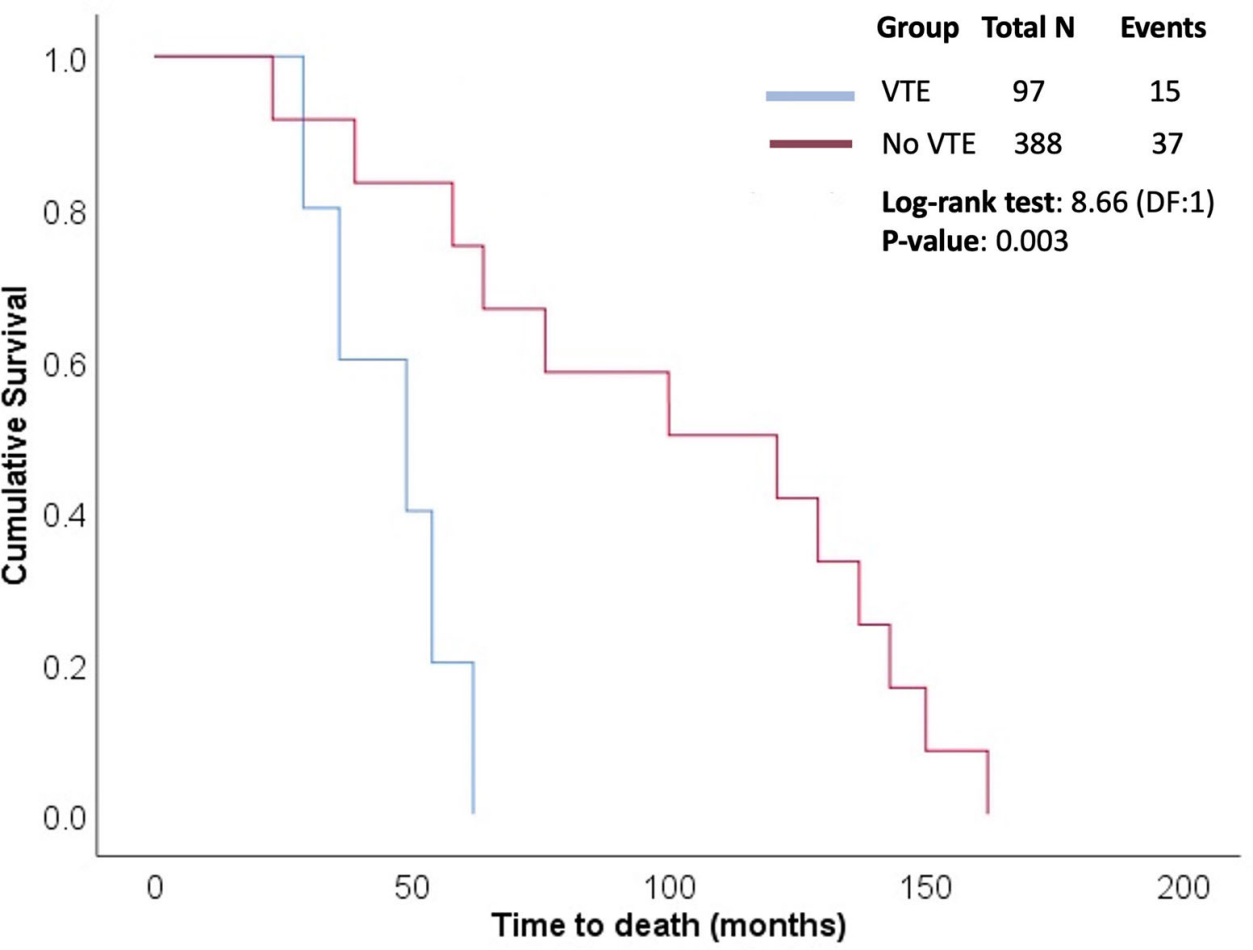
Kaplan-Meier plots showing cumulative survival rates in two study groups.

Of the 97 patients who experienced VTE, 15 (15.6%) died. Six patients with pulmonary thromboembolism died of acute massive episodes, five of lower respiratory tract infection, and four of myocardial infarction. Of the 388 patients without VTE, 37 (9.4%) died. Survival analysis was performed considering death as an event, and time to event was compared between two patient categories: VTE and no VTE. The estimated mean time to event in the VTE group was 46 months (95% CI: 34.26–57.74; SE: 5.99), with a median of 49 months (95% CI: 21.08–76.91; SE: 14.24). The Kaplan–Meier plot showing the cumulative survival in the two groups is presented in Figure 2. The difference in the survival rates between groups was statistically significant (*p* = 0.003), obtained using the Log-rank test. The patients experiencing VTE had significantly shorter survival time than patients without VTE.

**Figure 3.**
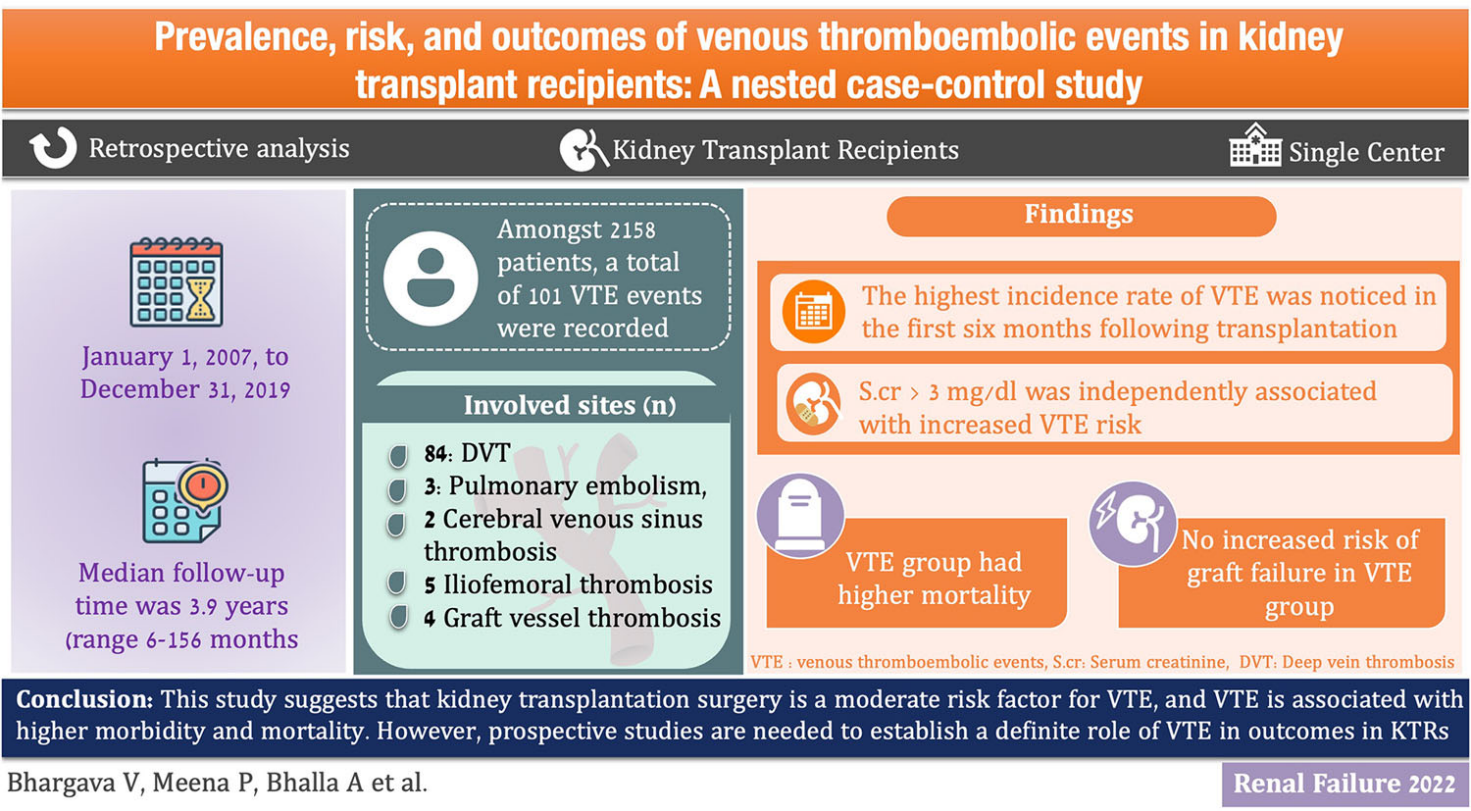
Graphical representation of the study.

### Effect of VTEs on graft loss

Of the 97 patients who experienced VTE, 9 (9.2%) had graft failure. The adjusted hazard ratio (HR) for graft loss in patients with VTE was 1.1 [95% confidence interval (CI): 0.5–3.1]. It was statistically not significant (*p* = 0.38). The HR was calculated after adjusting for confounders including acute rejection episodes, delayed graft function, recipient’s age, BMI and co-morbidities, and donor variables such as age and sex.

## Discussion

VTE is a well-established entity that develops commonly after various surgeries. Though, previous studies have examined VTE in patients with kidney diseases, the prevalence, risk factors and outcomes of VTE in KTRs are largely unrecognized. KTRs are considered a high-risk population for developing VTE due to multiple risk factors. Despite these facts, the impact of VTEs in KTRs and especially on patient and graft survival is not well described in the past literature. Thus, there is a lack of robust evidence on thromboprophylaxis strategies in these KTRs. Our study enrolled a large cohort of KTRs to affirm and study these unclear aspects. The present study not only evaluates the prevalence and risk factors of VTE but also determined its impact on graft and patient survival over a long follow-up period. In KTRs, the incidence of VTE complications varies from 0.6% to 25% [5, 6]. In the present study, the incidence of VTE events was 4.5%. In a retrospective cohort study from the United States National Registry, the rate of VTE 3 years after transplantation was reported to be 2.9 episodes/1000 person-years [2]. In another large series, the reported cumulative incidence of VTE after KT was 3.0%, 5.8%, and 8.4% at 1, 5, and 10 years, respectively. The study also reported the standardized incidence ratio (SIR) in KTRs as 7.9 (95% CI: 6.2–10.0) when compared with the general population [7]. The etiology of the hypercoagulable state in KTRs is multifactorial, including traditional risk factors that are similar in the general population, such as age, prolonged immobilization, malignancy, and postoperative state, and other risk factors specific to transplantation, such as immunosuppressive agents, including sirolimus and corticosteroids, increased inflammatory state, posttransplant erythrocytosis, heavy proteinuria, and sepsis [8]. We found that the risk of VTE was highest during the first 6 months post-transplantation. This observation is in line with previously published studies [9]. It is likely that the high dose of immunosuppressive agents and surgical factors predispose patients to VTE during this period. Several alterations in anticoagulant, procoagulant, and fibrinolytic pathways have been observed following KT. For example, there is a significant decrease in serum antithrombin, protein C, and thrombomodulin levels in the first 24 h after KT; moreover, there can be increased levels of fibrinogen and D-dimer [10]. In the present study, 84 (3.8%) patients had DVT and 6 (0.3%) had PE. Poli et al. reported both a lower limb DVT and PE incidence of 9.1% in KTRs [6]. Similarly, in another analysis by Kim Mi et al. of 829 KTRs, 7.2% developed VTE, with 49 cases (81.6%) of DVT [11]. The higher incidence of DVT in these reports compared with our study could be due to periodic screening and surveillance of asymptomatic cases, which did not occur in our analysis.

In graft vessels, there is localized activation of the coagulation system, leading to higher susceptibility to thrombosis. Thrombosis of allograft vessels is a well-known early complication in KTRs; it is mostly attributed to acute rejections, surgical complications, or hypovolemia [12]. Four of the cases in our study had allograft vein thrombosis, and all cases were due to acute rejection episodes. In one large series, 2.2% of KTs were complicated by graft thrombosis, with 90% of cases occurring within the first 2 weeks post-transplantation [13]. We analyzed the risk factors for VTE in KTRs and determined that a serum creatinine >3 mg/dl was associated with a higher risk of VTE. Our results were in line with another study that reported that eGFR <30 mL/min/1.73 m^2^ in the first year following KT was associated with an increased risk of VTE [2]. The higher risk of VTE in patients with low GFR could possibly be attributed to increased thrombogenicity due to impaired platelet function, oxidative stress, endothelial injury, inflammation, and hyperhomocysteinemia [14]. Studies have shown that the presence of severe proteinuria can predispose to higher VTE risk in KTRs, especially with recurrent glomerulonephritis following transplantation due to low antithrombin III and high fibrinogen levels [5]. However, our analysis found no association with higher VTE risk with nephrotic-range proteinuria. With and Kazory et al. reported seven cases of simultaneous acute CMV infection and VTE in KTRs [15]. However, we found no such association in our analysis. Other large analyses have also failed to report such a cause-and-effect relationship [2]. A recent study found an association between ABO blood groups and thrombophilia, however, our study found no such association [16]

Zannazi et al. reported a high risk of VTE recurrence in KTRs after the first episode and suggested prolonged anticoagulation for secondary thromboprophylaxis while being vigilant for bleeding complications [4]. Our study supports the findings of previously published studies that the occurrence of VTE is associated with higher mortality in KTRs [17]. Our results did not show an increased risk of graft failure in VTE patients. Analyses by Abualhassan N et al. showed that in patients with VTE, the risk of graft failure increased by 30%, but was statistically insignificant [18]. Currently, there is no consensus on screening recipients before KT for thrombophilia disorders; however, screening may be beneficial in patients who have a history of VTE, such as repeated thrombosis of vascular access.

This study had certain limitations. First, all the relevant investigations, including laboratory and radiological data, were analyzed retrospectively; therefore, there is a lack of independent verification. Second, being a single-centre analysis, the results of the study cannot be generalized. The availability of data was inconsistent for prothrombotic markers, such as factor V Leiden and serum homocysteine levels, in our study population; therefore, there is the possibility of confounding. Though convincing, findings were largely associative, and a causal conjunction could not be robustly established. Regular screening for DVT, even in asymptomatic patients, could have provided an accurate estimation of the VTE prevalence. Finally, the risk of VTE in our study population was not compared to the general population. Data on the exact etiology (biopsy proven) of ESKD could not be retrieved for all the cases, it could have added more value to the results. However, our study is the first from our country to report the prevalence of VTE in a large cohort with a long follow-up period (Figure 3).

Conclusions: In conclusion, the study indicates that VTE events are more common in KTRs with advanced renal impairment. It is most common in the early part of the post-transplant period. VTE has a significant impact on survival in KTRs. We foresee long-term randomized controlled trials to analyze the risk factors and formulate future screening, accurate diagnostic, and treatment approaches.

## Ethical approval

The study was reviewed and approved by the institutional review boards of the hospital.

## Informed consent statement

This is a retrospective analysis that is approved by the institutional review board. (EC/06/21/1943)

## Data Availability

There are no additional data available.
